# Validation of Ohmic Heating Pilot Plant for Vitamin C Retention and *E. coli* Surrogate Inactivation on Strawberry Nectar

**DOI:** 10.1155/ijfo/2464512

**Published:** 2025-10-08

**Authors:** Dario J. Pavon-Vargas, Vincenzo Alfonsi, Stephane Georgé, Mario Gozzi, Sara Rainieri, Luca Cattani

**Affiliations:** ^1^ Department of Engineering and Architecture, University of Parma, Parma, Italy, unipr.it; ^2^ Department of Food and Drug, University of Parma, Parma, Italy, unipr.it; ^3^ Centre Technique de Conservation des Produits Agricole, Avignon, France; ^4^ CFT S.p.A., Parma, Italy; ^5^ Department of Engineering for Industrial Systems and Technologies, University of Parma, Parma, Italy, unipr.it

**Keywords:** microbial inactivation, ohmic heating, pilot-scale, thermal kinetics, vitamin C

## Abstract

This study validates the performance of a pilot‐scale ohmic heating plant for vitamin C retention and *Escherichia coli* surrogate inactivation in strawberry nectar, based on thermal kinetics determined using a thermoresistometer. Initial experiments with the thermoresistometer established the thermal kinetics of vitamin C degradation and *E. coli* ATCC 8739 inactivation in surrogate media and strawberry nectar. The use of the thermoresistometer was selected due to its rapid heating mechanism, which closely matches the heating rates of ohmic heating. The *D* values for the microorganism ranged from 19.8 to 123.6 s, and the activation energy for vitamin C was 25.83 ± 0.48 kJ/mol for the surrogate media and 31.00 ± 2.62 kJ/mol for the nectar. Based on these results, treatments in the pilot‐scale ohmic heating system were designed to achieve a 5‐log microbial reduction and minimal vitamin C loss. The pilot trial on strawberry nectar demonstrated effective microbial inactivation and a reduction in vitamin C of 17%, which was higher than the calculated 2.7% from thermoresistometer data, likely due to differences in processing conditions. This research confirms that ohmic heating can achieve comparable microbial safety and nutrient preservation to conventional pasteurization, while offering potential advantages in energy efficiency.

## 1. Introduction

Ensuring microbial safety and maintaining the nutritional integrity of fruit‐based products are critical objectives in food processing. Ohmic heating (OH) is a novel technology that uses alternating current to heat food directly. Electrodes placed in the food send electric currents through it, causing even and quick heating. This process is faster, cleaner, and more environmentally friendly, helping to keep more of the food’s nutrients intact [1, 2]. It has been extensively studied and used in the industry for its effectiveness in pasteurizing fruit juices, resulting in minimal nutrient loss and preservation of the product’s sensory attributes [3, 4].

The US Food and Drug Administration (FDA) requires a 5‐log reduction of relevant food pathogens to ensure the safety of juice products like strawberry nectar (SN) [5]. Meeting this requirement is challenging, as it involves a process that can effectively inactivate harmful microorganisms while preserving the product’s nutrients and quality [6]. Achieving this balance is essential to meet both microbial safety standards and consumer demand for high‐quality, nutrient‐rich foods. SN is rich in vitamin C, a compound that is highly sensitive to heat [7]. Traditional thermal processing frequently causes undesirable changes in the quality of the final product, such as vitamin C degradation and alterations in color [8, 9]. Recent literature indicates that OH can significantly reduce the loss of thermolabile nutrients such as vitamin C, anthocyanins, and polyphenols during processing. Thus, the primary objective of OH is not only to ensure food safety but also to maintain the nutritional and sensory quality of the nectar [10].

Recent studies have established that *Escherichia coli* ATCC 8739 has emerged as a suitable surrogate for testing heat treatments in fruit nectar production [11], as it offers similar resistance to temperature as pathogens like *E. coli* O157:H7, which is a common cause of food‐borne disease outbreaks linked to fruit juices [12]. Therefore, the main objective of this study was to achieve the required reduction of the surrogate *E. coli* ATCC 8739 while simultaneously evaluating the retention of vitamin C using a pilot‐scale unit.

Scaling up the OH process to a pilot plant level is essential to fully assess its effectiveness in retaining vitamin C and inactivating pathogens in SN. OH generally allows for uniform electricity distribution, leading to consistent and even heating across the product. This uniformity simplifies the scale‐up process, minimizing many of the challenges typically encountered when transitioning from lab scale to pilot or industrial scale [4]. Nevertheless, at larger scales, challenges related to uniform heating, energy efficiency, and nutrient retention become more complex, and the validation process is critical to ensure consistent results and viability for industrial applications. Consequently, validating the process at pilot scale becomes essential for assessing its potential for nutrient retention and microbial safety in commercial applications.

Considering that both thermal and ohmic methods involve heat‐based processing, a comparison between them is essential, as both are thought to share similar degradation mechanisms [1]. Moreover, literature presents conflicting results regarding the degradation of bioactive compounds during OH, particularly in relation to the effects of the electric field. Some studies suggest that equivalent treatment times can lead to greater preservation of bioactive compounds compared to thermal methods, while others report similar or even increased degradation rates, possibly due to variations in electrode material or electric field strength [13, 14].

While previous research often focused on microbial reduction or nutrient retention independently and usually on a laboratory scale, this study attempts to bridge these gaps by adopting a synergistic approach, aiming to evaluate both critical factors, microbial inactivation and nutrient retention, in a pilot‐scale environment. This investigation not only addresses the need for more comprehensive validations of emerging technologies; it also broadens our understanding of how to optimize OH, particularly in minimizing bioactive compound degradation.

In this research, for the kinetic measurements, the thermoresistometer was selected for its ability to rapidly achieve heating rates comparable to those of OH, which allowed us to approximate the thermal effects expected in the pilot‐scale ohmic system.

## 2. Materials and Methods

### 2.1. Reagents and Experimental Solutions

In this study, HPLC‐grade water from Thermo Fisher, anhydrous citric acid from Fisher BioReagents (≥ 99.5%), and HPLC‐grade acetonitrile from Acros Organics were used. All other analytical grade chemicals were purchased from Sigma‐Aldrich. The ascorbic acid (AA) solution was prepared by dissolving 5 g of l‐AA in 25 mL of 2.31% acetic acid, resulting in a final concentration of 200 g/L. The solution was stored at 4°C until each lab trial. McIlvaine’s buffer solution (BS), with an initial pH of 3.5, was used as a model solution (MS) during the lab‐scale trials; it was prepared by mixing 0.1 M citric acid and 0.2 M disodium phosphate solutions [15]. To achieve the final pH of 3.14, a 5% v/w citric acid solution was gradually added until the desired pH was reached. The BS was then stored at 4°C until use. SN was prepared using frozen untreated strawberry puree (SP) (*Fragaria x ananassa*), purchased from SVZ International (Breda, The Netherlands). Sucrose and citric acid were added to the puree, along with filtered tap water. The final formulation contained 40% (w/w) strawberry puree, sucrose added to achieve 12% total soluble solids (TSSs), and citric acid to reach an acidity level of 5.0 g/kg, resulting in a final pH of 3.12 ± 0.02. Additionally, a MS was formulated to minimize the use of SP during the pilot trials, which would require approximately 400 L/h. This solution replicated the conductivity, vitamin C concentration, and pH of SN. It contained citric acid, sucrose, and AA. The pH was adjusted to 3.14 using a 1 M disodium phosphate solution, and the conductivity was adjusted by adding an appropriate amount of salt (NaCl).

### 2.2. Vitamin C Analysis

The concentration of AA in the samples was measured using a modified version of the procedure by [16]. Five grams of sample was mixed with 50 mL of 4% metaphosphoric acid (MPA). To quantify both AA and dehydroascorbic acid, a reduction step was performed using tris(2‐carboxyethyl)‐phosphine hydrochloride (TCEP). The solution was then filtered through a Whatman cellulose acetate (CA) syringe filter (0.45 *μ*m) and transferred to HPLC vials for analysis.

Vitamin C analysis was carried out using an Agilent 1260 Infinity LC system (Agilent Technologies, Waldbronn, Germany) coupled with a 1290 Agilent diode array detector (DAD) from Agilent Technologies. A C18 ACE column (250 × 4.6 mm, 5 *μ*m particle size) was employed. The mobile phase consisted of 100%, 0.01% sulfuric acid (0.1 mL of concentrated sulfuric acid in 1 L of HPLC‐grade water), with a flow rate of 0.8 mL/min. The column temperature was maintained at 30°C, and 100 *μ*L of each sample was injected for analysis. Data acquisition was conducted at 245 nm. Quantification was performed using a calibration curve based on AA standards, and results were reported as total AA content.

### 2.3. Inoculum Preparation and Microbial Analysis

Microbial inactivation was evaluated using *E. coli* ATCC 8739 as a surrogate pathogen, following the procedure by [17] for growth and maintenance. A frozen stock culture of *E. coli* ATCC 8739 was thawed and reactivated in tryptic soy broth (TSB) at 37°C for 16 h. This reactivated culture was then diluted in acidified TSB to match the pH of the nectar and incubated for an additional 24 h at 37°C, resulting in an initial concentration of approximately 10^9^ CFU/mL. This prepared inoculum was subsequently used for the microbial analysis in lab‐scale trials.

For each test condition, microbial samples were serially diluted and plated on tryptic soy agar (TSA), then incubated at 37°C for 24 h to allow colony formation for viable count determination.

### 2.4. Vitamin C Reduction Kinetics—Lab Scale

The kinetics of vitamin C reduction were analyzed using a Mastia thermoresistometer (Polytechnic University of Cartagena, Cartagena, Spain) in a 400‐mL stirred vessel operating in isothermal mode. The system and operational setup, as described by Conesa et al. [18], allowed for uniform and fast heating of the media to specific temperatures (65°C, 85°C, and 105°C). After heating the solution to the selected temperature, 600 *μ*L of the AA solution was added to achieve a final concentration of 30 mg/100 mL in the BS. The mixture was then shaken at a speed of 1950 rpm while maintaining the target temperature. The treatment lasted 180 min, with samples collected at specific intervals: every 5 min for the first 30 min, every 10 min from 30 to 60 min, and every 30 min from 60 to 180 min. Each sample was filtered and analyzed using HPLC. This entire treatment process was repeated three times for each temperature. For comparative analysis of degradation rates, BS was evaluated under the same conditions as the SN.

The degradation of AA was evaluated using first‐order kinetics [13]. The first‐order model is described by

(1) AA=AA0·exp−k·t

where [AA] is the AA concentration (mg AA/100 mL) at time *t*, [AA]_0_ is the AA concentration at time 0, and *k* is the AA degradation rate constant for the first order (s^−1^). The temperature dependence of AA degradation was expressed using the activation energy (*E*_*a*_) described by Arrhenius kinetics.

### 2.5. Microbial Inactivation Kinetics—Lab Scale

As part of the project, kinetic studies on the nectar with the same surrogate were previously conducted [11]. To corroborate these findings and ensure validation, selected data points were analyzed both in BS and SN. Further validation was performed in the MS in subsequent stages. Microbial inactivation was performed on the thermoresistometer for both the BS and the SN. The prepared inoculum was injected into the thermoresistometer vessel at a 1:100 dilution, resulting in an approximate final concentration of 10^7^ CFU/mL. Before inoculation, both SN and BS were sterilized by heating to 145°C for 2 min. After sterilization and inoculation, the solutions were heated to the target temperature (60°C, 65°C, and 70°C). Once the target temperature was reached, viable counts were measured at specific intervals (0, 0.5, 1, and 2 min) to assess the rate of microbial inactivation. In this assay, BS was adjusted to 12% TSSs by adding sucrose to match the composition of SN. Samples were taken at the specified intervals, and microbial load was counted as previously described.

The inactivation of *E. coli* ATCC 8739 was modeled using the Bigelow kinetics, which assumes first order [19]. This approach was applied to both the SN and BS. The model is expressed by

(2) Nt=N0·exp−k·t

where *N*_*t*_ represents the number of microorganisms at time *t*, *N*_0_ is the initial number of microorganisms, and *k* is the first‐order rate constant (s^−1^). This equation can be rearranged to relate the *k* constant to the decimal reduction time *D* by

(3) D=2.303k.

The temperature dependence of *D* is described by the *z* value, and the *z* value can be related to *E*_*a*_ by

(4) z=2.303·R·T2Ea.

### 2.6. Initial Physicochemical Parameters

The initial parameters were evaluated in both the SP and the SN and later used to calculate and adjust the MS to match the values. pH was measured at 20°C using a FiveEasy pH/mV meter (Model F20, Mettler Toledo, Shanghai, China). The electrical conductivity was measured using a fixed‐volume cell (10 × 10 × 25 cm) with two metallic plates at each end. A fixed current was applied, and the resulting voltages were measured with the cell empty and with the product present, and conductivity was calculated by applying an empirical relationship that incorporates both the cell constant and the generator constant. TSSs were determined using a digital refractometer (DBX‐55, Atago, Tokyo, Japan), and acidity was measured by potentiometric titration with 0.1 N NaOH solution using a Metrohm 916 Ti‐Touch titrator (Metrohm, Herisau, Switzerland). Acidity was expressed as citric acid.

### 2.7. Sample Preparation and Inoculation

The preparation of SN and MS was completed on the same day as the pilot trials. For SN, previously frozen SP was thawed over 2 days. The SN and MS were then prepared by mixing the specified ingredients in calculated quantities, resulting in 400 L of SN and 300 L of MS. Before processing, the SN was preheated to 20°C in a heating and mixing tank to match the initial temperature conditions used in the MS trials. Inoculum preparation for the pilot trials began 3 days prior. On the 1st day, the culture was diluted to 1:100, reaching a volume of 30 mL. This was expanded to 300 mL on the 2nd day and further increased to a final volume of 3 L on the 3rd day, achieving an approximate concentration of 10^9^ CFU/mL. On the treatment day, 3 L of inoculum was introduced into 300 L of MS for a final concentration of approximately 10^7^ CFU/mL. Laboratory analysis confirmed an actual initial load of 9.33 × 10^6^ CFU/mL (6.97 log CFU/mL). The pilot trials were conducted with two replicates for each condition, performed over two consecutive weeks, with one trial per week. The pilot‐scale validation was specifically designed to assess the influence of the electric field in OH, which is not replicated by the thermoresistometer. This approach enabled us to compare the thermal kinetics from the thermoresistometer with the actual performance of the OH pilot plant, thereby providing a comprehensive evaluation of the technology’s effectiveness.

### 2.8. OH System

**Figure 1:**
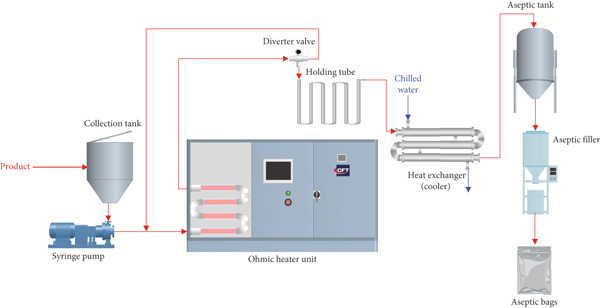
Schematic diagram of the pilot‐scale continuous ohmic heating system and associated equipment.

The OH experiments were conducted using a continuous three‐phase pilot‐scale system (CFT S.p.A., Parma, Italy) installed at the Centre Technique de la Conservation des Produits Agricoles (CTCPA) in Avignon, France. Three independent 20‐kW generators (totaling 60 kW) supplied the system, operating at a voltage range of 1–5 kV and a frequency of 25 kHz. The system comprised three pairs of Schott DURAN SP9mm glass tubes arranged in parallel, each with an internal diameter of 32 mm and a length of 220 cm. These tubes were connected by U‐shaped unions, such that a set of these assemblies were used per generator line. Stainless steel electrodes (AISI 316L) were arranged in an annular configuration at each pass. Thermocouples were installed at the end of each phase, allowing automated and accurate monitoring and regulation of the product temperature to the set point. The product, either MS or SN, was pumped from a collection tank via a syringe pump into the OH unit at a flow rate of 380 L/h. After passing through the ohmic chamber, the product was directed by a divert valve to a 9‐L holding tube for sufficient residence time, followed by rapid cooling in a closed aseptic heat exchanger using chilled water to lower the temperature below 20°C; the treated product was then stored in an aseptic tank before aseptic filling. The filled product was packaged into metallized Fres‐co aseptic system bags (Goglio S.p.A., Milan, Italy) using a Macropack F1 aseptic filler (CFT S.p.A., Parma, Italy). The system can achieve a heating rate of 6°C/s in stationary mode. A schematic diagram of the pilot‐scale OH system is presented in Figure 1.

For the MS trials, processing was conducted at 65°C with a 3‐min holding time to achieve a 5‐log microbial reduction, based on previously calculated kinetics. Both vitamin C degradation and microbial inactivation were monitored, with untreated samples collected as controls. Following the MS trials, the SN was processed at 85°C for 2 min in the same system. Samples were collected in triplicate for vitamin C analysis and in duplicate, plated on duplicate plates, for microbial analysis.

### 2.9. Data Analysis

Statistical differences were assessed using Student’s *t*‐test, with Levene’s test to confirm homogeneity of variance. A significance level of *p* = 0.05 was used to determine statistical significance. Hypothesis testing was conducted to compare the mean values of the experimental data with the modeled values. *p* values and standardized residuals were calculated to evaluate significant deviations from model predictions. Additionally, models were evaluated based on their coefficient of determination (*R* ^2^) values. All statistical analyses were performed using IBM SPSS software Version 29.0.1.0, while regression analysis was carried out in Microsoft Excel.

## 3. Results and Discussion

### 3.1. Kinetics of Vitamin C Reduction and Microbial Inactivation

Table 1 presents the kinetic parameters for the degradation of AA and the reduction of *E. coli* ATCC 8739 in both SN and MS, providing a comparison of their behavior under the conditions tested. The degradation of vitamin C in both SN and BS was evaluated using first‐order kinetics [20]. No significant changes in thermal kinetic parameters are expected between the SN and the BS or MS when pH and brix are matched, as these are the primary drivers of thermal inactivation kinetics. Minor differences may arise from the presence of additional bioactive compounds in the nectar matrix, but these are not expected to substantially alter the overall kinetic behavior in the context of this study [11, 21, 22]. The results showed that temperature played a crucial role in the degradation rate, with higher temperatures leading to accelerated loss of vitamin C (higher *k* value). The first‐order model provided a clear description of the degradation (*R*‐squared > 0.98), indicating a strong representation of the degradation process. The Bigelow kinetic parameters were calculated from the previously determined *k* and *E*_*a*_ values, enabling the use of both kinetic models in the predictive modeling prior to the pilot trials.

**Table 1:** Kinetic parameters for the degradation of ascorbic acid and the reduction of *E. coli* ATCC 8739 in strawberry nectar (SN) and buffer solution (BS).

**Parameter**	**Matrix**	**T (°C)**	**k** **(s** ^**−1**^)	**E**_**a**_ **(kJ/mol)**	**D** **(min)**	**z** **(°C)**	**R** ^2^
Vitamin C	BS	65	0.92 ± 0.21	25.83 ± 0.48	150.7 ± 13.35	93.81 ± 1.74	0.963
		85	2.54 ± 0.57		54.49 ± 12.91		0.971
		105	4.57 ± 0.21		30.24 ± 2.62		0.978
	SN	65	0.51 ± 0.05	31.00 ± 2.62	271.5 ± 10.04	80.10 ± 6.77	0.912
		85	0.96 ± 0.02		143.31 ± 2.17		0.923
		105	1.16 ± 0.04		118.72 ± 3.61		0.937

*E. coli* ATCC 8739	BS	60	0.024 ± 0.001	150.54 ± 2.80	1.62 ± 0.08	14.54 ± 0.27	0.959
		65	0.065 ± 0.001		0.59 ± 0.01		0.865
		70	0.116 ± 0.002		0.33 ± 0.01		0.857
	SN	60	0.019 ± 0.001	108.36 ± 3.27	2.06 ± 0.03	20.20 ± 0.61	0.991
		65	0.033 ± 0.002		1.17 ± 0.06		0.978
		70	0.058 ± 0.001		0.66 ± 0.01		0.823

Although the results for SN and BS were generally comparable, there was a noticeable deviation. This suggests that while the degradation rate (*k* value) of vitamin C in the nectar may be slower, it is more sensitive to temperature changes (*Z* value). Additionally, when plotting rate constants (*k*) for both the BS and SN processes against the reciprocal of absolute temperature to calculate the frequency factor (*k*_0_), the degradation kinetics of SN showed a higher factor (*k*_0_ = 0.98 s^−1^) compared to the SP (*k*_0_ = 0.31 s^−1^). The higher *k*_0_ value observed in SN compared to BS suggests that, even at comparable temperatures, the intrinsic rate of degradation reactions in the nectar is higher, likely due to its more reactive or catalytically active environment. This is probably due to the different matrix composition and the nature of AA in the nectar versus the solution, affecting heat transfer and degradation behavior. The degradation of compounds is generally faster in real matrices compared to model systems [23, 24]. In the study by Van Bree et al. [25], vitamin C degradation kinetics present higher *k* values in imitation fruit juice compared to commercial juice, but the model accurately predicted the behavior. Therefore, despite this variation, the BS still provides a reasonable approximation, supporting its use for simulating the nectar during OH.

The kinetic parameters of vitamin C degradation observed in this study are consistent with those reported in the literature for thermal degradation in strawberry products [7, 26]. While the results are focus on thermal degradation kinetics, it is important to note that studies comparing ohmic and conventional heating on strawberries indicate similar degradation behaviors for AA in both methods [27, 28]. For example, Castro et al. reported rate constants (*k*_0_) of 0.15 s^−1^ with activation energies (*E*_*a*_) of 21.36 kJ/mol, with no statistically significant differences observed between ohmic and thermal treatments [28]. This suggests that the electric field during OH does not significantly alter the degradation pathway of vitamin C, as any substantial impact would likely change the activation energy. Literature further suggests that similar kinetics can be achieved with industrial equipment, where the use of specific electrode materials and higher frequencies minimizes corrosion effects on degradation [13]. Accordingly, industrial‐grade equipment and high‐frequency settings were later used in the pilot trials.

Microbial inactivation was modeled using Bigelow kinetics. The *D* and *z* values for *E. coli* ATCC 8739 were calculated in both SN and BS. The results indicated that *E. coli* inactivation followed a first‐order model, with faster inactivation observed at higher temperatures, as expected. The reaction kinetics and activation energy (*E*_*a*_) were calculated from the Bigelow kinetic parameters. The microbial inactivation kinetic parameters in both SN and BS were comparable, confirming that the MS can represent the inactivation behavior of the nectar. The *z* values were 14.54 and 20.02 for the MS and SN, respectively, indicating high thermal resistance.

In literature, *D* values for *E. coli* at 60°C range from 13.2 s to 5 min, which is in good agreement with our findings [29]. A study on different strains of *E. coli* surrogates, including ATCC 8739, reported *D* values between 13.8 and 99.6 s and *z* values between 19.66 and 24.89°C in the same temperature range for different fruit nectars [11]. Similarly, another study reported a *D*_50_ of 324.6 s, a *D*_60_ of 95.4 s, and a *z* value of 18.78 ± 1.44^°^C for *E. coli* O157 in orange juice. These comparable results further validate the use of *E. coli* ATCC 8739 as an appropriate surrogate for heat treatments in acidic fruit–based products like SN.

The selection of 65°C and 3 min treatment of the MS for the pilot trials was guided by kinetic parameters aimed at achieving a 5‐log reduction of *E. coli* while minimizing vitamin C loss. Additionally, the equipment imposes limitations on flow rate; achieving higher temperatures would require a significantly faster flow rate, which may not be feasible. Typically, thermal pasteurization in juices results in a vitamin C loss of a minimum of 5% [30]. Nevertheless, different heating methods, even at similar temperatures, can result in different levels of vitamin C degradation [31]. According to the model, the 5‐log reduction in *E. coli* at 65°C corresponds to a treatment time of 3 min, which was further validated during the pilot scale trials. For SN, a temperature of 85°C with a 2‐min holding time was chosen, based on the specific kinetics of SN vitamin C degradation and microbial inactivation, as well as alignment with common thermal treatment practices. Similar temperature–time combinations are frequently used in thermal pasteurization of fruit‐based products [6].

Figure 2 presents the mathematical predictions using the Bigelow model for achieving a 5‐log reduction of *E. coli* ATCC 8739 (dashed lines), alongside the modeled degradation of AA (dot‐dashed lines) for both the MS (Figure 2a) and SN (Figure 2b). In Figure 2a, the blue dot‐dashed line represents a 9% loss of AA, which aligns with the experimental treatment point of 65°C for 3 min. Similarly, Figure 2b shows the modeled AA degradation curve (green dot‐dashed line), corresponding to a 3.0% loss. This curve intersects with the treatment point chosen for SN at 85°C for 2 min, which resulted in a significantly higher microbial reduction. The treatment for SN was selected to validate the model, considering the kinetics of AA in SN, the limitations of the flow rate in the pilot unit, and the raw material available.

Figure 2Temperature–time chart for mathematical predictions (Bigelow model) of 5‐log *E. coli* ATCC 8739 reduction and ascorbic acid loss in (a) model solution and (b) strawberry nectar at selected treatment points.

**Figure:**
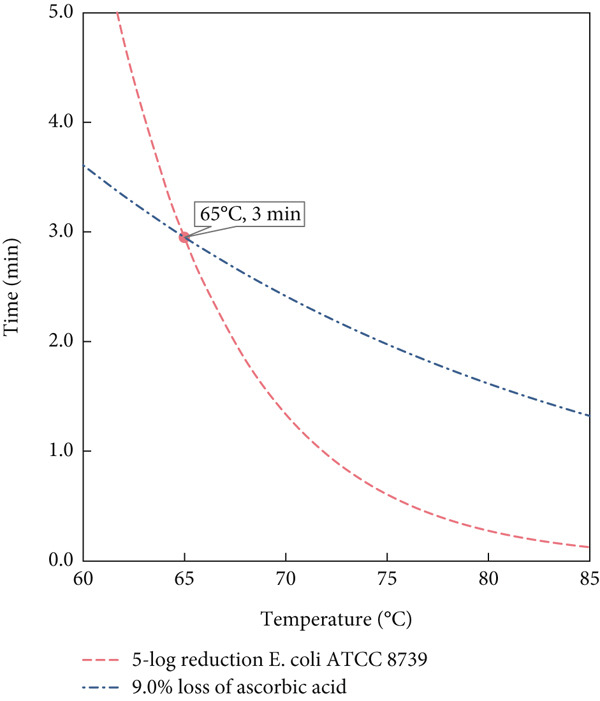
(a)

**Figure:**
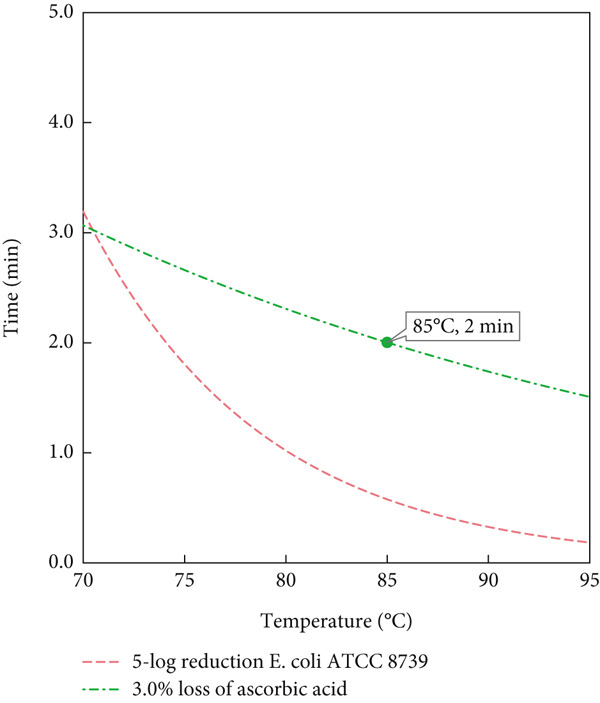
(b)

### 3.2. Initial Physiochemical Parameters

The physicochemical parameters of the SP, SN, and MS are presented in Table 2. These parameters include pH, TSS, electrical conductivity, AA content, acidity, and the initial microbial count. Electrical conductivity is an important parameter for the OH process, as it directly affects the efficiency of heat generation [32]. The MS was specifically designed to replicate the electrical conductivity of the SN as closely as possible to ensure accurate comparison. However, it is important to acknowledge that other properties of the MS, such as heat capacity and viscosity, may cause variations in heating behavior compared to the actual nectar. Different heat capacities between MS and SN mean they will absorb and retain heat at different rates, and viscosity may affect the fluid’s flow and distribution of heat within the system.

**Table 2:** Physicochemical properties of strawberry puree (SP), strawberry nectar (SN), and model solution (MS) with *p* values from *t*‐test comparison between SN and MS.

**Properties**	**SP**	**SN**	**MS**	**p** **value**⁣^∗^
Total soluble solids (TSSs) (°Brix)	7.9 ± 0.1	11.9 ± 0.1	11.8 ± 0.1	0.553
pH at 20°C	3.40 ± 0.01	3.16 ± 0.02	3.14 ± 0.03	0.519
Acidity as citric acid at 25°C (g/kg)	7.49 ± 0.08	5.23 ± 0.11	5.15 ± 0.04	0.448
Electrical conductivity at 20°C (mS/cm)	—	1.07 ± 0.03	1.04 ± 0.07	0.675
Total ascorbic acid (mg/100 g)	89.6 ± 0.2	34.2 ± 1.0	36.0 ± 1.2	0.242
Total aerobic count (log CFU/mL)	4.92	2.31	< 1	—

^*^Two‐sample *t*‐test confidence interval of 95%. *p* < 0.05 indicates significant differences between strawberry nectar (SN) and model solution (MS).

As it can be seen in Table 2, there were no statistically significant differences between the physicochemical parameters of the SN evaluated at the laboratory level and the MS used at the pilot scale. The pH, TSS, and electrical conductivity values were particularly similar, supporting the validity of using the MS to simulate the behavior of the actual nectar during OH. The data from these laboratory evaluations provide the basis for comparing the thermal and OH responses of the different matrices, ensuring that the model system could accurately represent the nectar.

The physicochemical values for the SP and SN, with pH values between 3.1 and 3.4, and AA content of 63.6–89.0 mg/100 g for the puree and 39.8 ± 1.1 mg/100 g for the nectar are consistent with previous studies [12, 33, 34].

### 3.3. Pilot‐Scale Trials With MS

**Figure 3:**
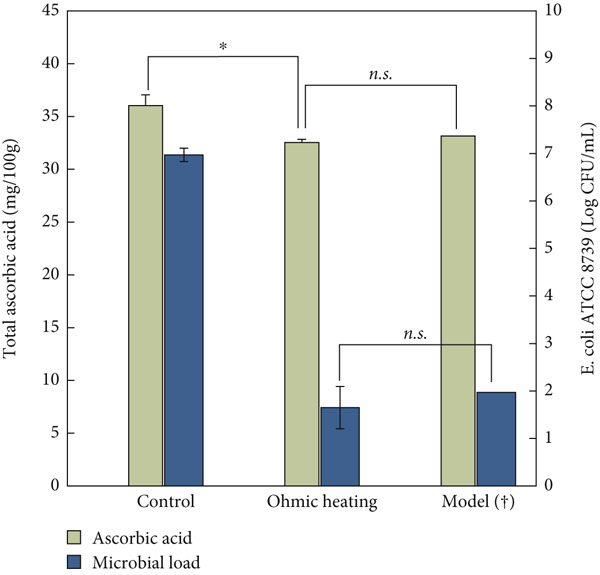
Comparison of ascorbic acid content (mg/100 g) (left axis) and microbial load (*E. coli* ATCC 8739) (log CFU/mL) (right axis) after ohmic heating at 65°C for 3 min, in a model system. Bigelow kinetic model predictions (†), control (before treatment), and ohmic heating (after treatment). Statistical differences (*p* < 0.05) are marked by asterisk (⁣^∗^), and *n.s.* denotes no significant difference.

During the pilot‐scale OH trials, the MS was processed to assess both vitamin C degradation and microbial inactivation. The results from these trials were consistent with those observed in the thermoresistometer experiments. The model, which was derived from Bigelow kinetics for a 5‐log reduction in *E. coli* and a 9% loss of AA, provides the basis for comparison with the pilot‐scale OH results. As can be seen in Figure 3, the comparison between the experimental results from OH and the model‐based kinetics (†) shows no significant differences in AA retention and microbial inactivation, aligning with the model predictions. The comparison between the laboratory‐scale and pilot‐scale trials showed that the OH system maintained similar kinetic behavior, supporting the scalability of the process.

As shown in Figure 3, the reduction after thermal treatment at 65°C for 3 min resulted in a significant reduction of 9.71%, which is in good agreement with the expected results from the model. A two‐sample *t*‐test comparing the ohmic‐treated samples with the modeled reduction (derived from the mean control value) shows a *t*‐statistic of −1.44 and a *p* value of 0.287, indicating no significant difference between the two. The vitamin C content in the ohmic‐treated sample was 32.53 ± 1.02 mg/100 g, while the model predicted a value of 33.14 mg/100 g. The similarity in kinetics between the lab‐scale thermal and pilot‐scale OH trials with the MS suggests that, despite the differences in heating mechanisms, both systems achieved comparable heat transfer effects.

In the lab‐scale thermal setup, heat transfer occurs through conduction and convection from an external source, resulting in a fast and uniform temperature increase across the solution, and the continuous stirring enhances the uniformity. In pilot‐scale, OH generates heat internally, as the electric field directly heats the MS [35]. In a uniform simple product like the MS, the electric field distributes evenly, closely mirroring the uniformity achieved with the thermoresistometer.

In terms of microbial validation, no significant difference was found between the model and the ohmic‐treated samples (*p* value of 0.343). The log reduction for the ohmic treatment was 5.32, compared to the model’s predicted value of 5. These results suggest that the model can accurately describe the behavior of *E. coli* surrogates and vitamin C reduction in the pilot unit, indicating that the kinetics of thermal treatments closely match those observed in OH. This corroborates the findings from other studies which have demonstrated similar kinetics for these processes [3, 13, 26].

### 3.4. Pilot‐Scale Trials With SN

**Figure 4:**
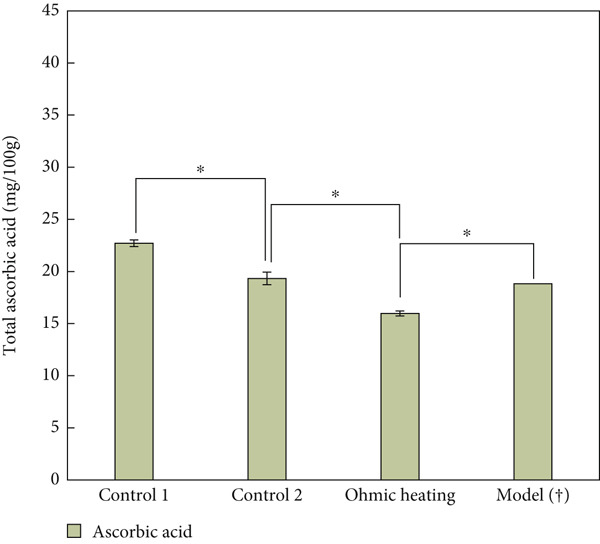
Comparison of ascorbic acid content (mg/100 g) in strawberry nectar treated at 85°C for 2 min in ohmic heating plant. Control 1 (immediately after mixing), Control 2 (just before ohmic processing), ohmic heating, and model predictions based on Bigelow kinetics (†). Statistical differences (*p* < 0.05) are marked by asterisk (⁣^∗^), and *n.s.* denotes no significant difference.

Figure 4 shows the comparison of AA content (mg/100 g) in SN treated at 85°C for 2 min in an OH plant. In this figure, AA retention is compared across Control 1 (immediately after mixing), Control 2 (just before ohmic processing), OH, and model predictions based on Bigelow kinetics. Control 1 and Control 2 were measured to assess not only the effect of ohmic processing but also the impact of the overall pilot plant operation on vitamin C levels. In the pilot trials with actual SN, the results showed a 19.45% difference in microbial reduction compared to the values predicted by the modeled system and thermal kinetics observed in the laboratory, indicating some inconsistency between the two.

An important observation is the lower AA content in Control 1 (22.71 mg/100 g), which is considerably lower than the lab‐measured value of 36.03 mg/100 g. This discrepancy may be attributed to the extensive defrosting of the nectar prior to processing, which could have led to the degradation of vitamin C. Additionally, industrial factors such as exposure to oxygen and prolonged residence in tanks could further contribute to this lower initial value. Control 2, sampled just before OH, showed an additional loss in AA compared to Control 1. This suggests that handling processes in the pilot plant have a cumulative effect on nutrient degradation.

As shown in Figure 4, the OH process resulted in a statistically significant lower retention of AA than expected, probably due to factors such as preprocessing losses and time in the aseptic tank or packaging. The Bigelow kinetic model predicted a higher retention; there is a 15% difference when comparing the model value to the ohmic‐treated samples, highlighting that models may not fully account for pilot conditions with real products. While the model predicted a reduction of 2.7% (to 18.81 mg/100 g), the actual degradation after treatment was 17.38%, yielding 15.97 mg/100 g. Overall, a 29.64% loss of AA occurred from the fresh nectar (Control 1). In the literature, the degradation of vitamin C during OH ranged from 3.08% to 10.63% [36], and in orange juice (12°Brix), conventional heating caused 22.45% degradation, while OH resulted in 20.03% [37].

OH can lead to electrolysis and electrode corrosion, increasing oxygen levels and accelerating AA oxidation. However, using stainless steel electrodes significantly reduces this effect [28]. Studies have shown that at frequencies above 100 Hz, the rate constants for conventional and OH are similar, suggesting that the electrical effect in ohmic has a minimal effect on the samples, with oxidation and temperature being the primary degradation mechanisms [10, 26]. OH may cause electrolysis and electrode corrosion, which can increase oxygen and accelerate AA oxidation. However, the use of stainless steel electrodes minimizes this effect [26, 27]. As industrial stainless steel electrodes operating at 25 kHz were used in this experiment, no electrolytic effect was expected; therefore, oxidation and thermal effect were the main degradation mechanisms.

## 4. Conclusions

The objective of this study was to validate an OH pilot plant for the retention of vitamin C and the inactivation of an *E. coli* surrogate in SN, using thermal kinetics as a predictive model. The pilot‐scale validation of the MS aligned with the thermal kinetic model calculated in the laboratory. The 5‐log reduction of *E. coli* was achieved at 65°C for 3 min, in line with the kinetic calculation, supporting the scalability of the process for industrial applications. In the SN test, however, OH resulted in more AA degradation than predicted, potentially due to factors like localized overheating, nonuniform energy distribution, and other variables inherent to pilot‐scale trials. These deviations highlight limitations in controlling conditions at pilot scale compared to laboratory setups, yet vitamin C degradation remains within the range of conventional thermal treatments. Additionally, the observed variability in AA retention underlines the challenges of scaling up from laboratory to pilot conditions. Future work should include pilot‐scale microbial validation specifically on SN to confirm the robustness and applicability of the process for real‐world fruit juice processing. Future studies could also explore optimizing electric field parameters and energy distribution to reduce variability and further preserve bioactive compounds. Expanding research across different matrices and process conditions could provide greater insights into the potential and adaptability of OH for diverse industrial applications. These improvements and validations will be essential for ensuring both food safety and nutritional quality in commercial‐scale operations.

## Conflicts of Interest

The authors declare no conflicts of interest.

## Author Contributions

Conceptualization: D.J.P‐V., V.A., M.G., and L.C.; methodology: D.J.P‐V., V.A., and S.G.; validation: D.J.P‐V.; formal analysis: D.J.P‐V. and V.A.; investigation: D.J.P‐V. and V.A.; resources: S.G., M.G., S.R., and L.C.; data curation: D.J.P‐V. and L.C.; writing—original draft preparation: D.J.P‐V.; writing—review and editing: M.G. and L.C.; visualization: D.J.P‐V.; supervision: S.G., S.R., L.C., and M.G.; project administration: S.G., M.G., S.R., and L.C.; funding acquisition: M.G.

## Funding

The project has received funding from the European Union’s Horizon 2020 research and innovation program under the Marie Sklodowska‐Curie grant agreement No. 956257.

## Data Availability

The data that support the findings of this study are openly available in Zenodo at https://doi.org/10.5281/zenodo.13938957.
